# Association Between Toothbrushing Habits and COVID-19 Symptoms

**DOI:** 10.1016/j.identj.2022.07.011

**Published:** 2022-08-04

**Authors:** Hazem Abbas, Kenji Takeuchi, Shihoko Koyama, Ken Osaka, Takahiro Tabuchi

**Affiliations:** a Department of International and Community Oral Health, Tohoku University, Graduate School of Dentistry, Sendai, Japan; b Osaka International Cancer Institute, Cancer Control Center, Osaka, Japan

**Keywords:** Toothbrushing, Oral hygiene, COVID-19, SARS-CoV-2, Dentistry, Public health

## Abstract

**Objectives:**

The association between toothbrushing and coronavirus disease 2019 (COVID-19) infections is unknown. The aim of this study was to test the hypothesis that the change in time and frequency of toothbrushing is associated with having COVID-19 symptoms.

**Methods:**

In this 8-month retrospective cohort study, we used the data from the Japan COVID-19 and Society Internet Survey (JACSIS; N = 22,366), which was conducted between August and September 2020. The logistic regression analyses were used to calculate the odds ratios (ORs) of having the 3 main COVID-19 symptoms (high fever, cough, and taste and smell disorder). Confounders were age, sex, educational attainment, equivalised income level, self-rated health, health literacy, and living area.

**Results:**

The mean age of the participants was 49 years (SD = ±17.3), and 49.2% were male. Overall 2704 (12.1%) participants changed (increased or decreased) the time and frequency of toothbrushing, whilst 19,662 (87.9%) did not change. Only 60 participants (0.3%) had the 3 main COVID-19 symptoms. All logistic regression models showed that those who had a change in time and frequency of toothbrushing had higher odds of having the 3 main COVID-19 symptoms compared to those who had unchanged time and frequency of toothbrushing. The ORs ranged from 6.00 (95% confidence interval [CI], 3.60–9.99) in the crude model to 4.08 (95% CI, 2.38–6.98) in the fully adjusted model.

**Conclusions:**

The change in time and frequency of toothbrushing from before to after the COVID-19 pandemic was associated with having the 3 main COVID-19 symptoms.

## Introduction

Since the declaration of the coronavirus disease 2019 (COVID-19) caused by the severe acute respiratory syndrome coronavirus 2 (SARS-CoV-2) as a pandemic in March 2020 by the World Health Organization (WHO),^1^ standard infection preventive measures such as wearing masks and social distancing were advised by health organisations worldwide.^2^ However, some oral health experts suggested that proper oral hygiene measures such as regular toothbrushing might play a role in the prevention of COVID-19 infection.^3^^,^^4^

Previous research showed that the oral cavity is an important site for SARS-CoV-2 viral replication, and the saliva could be a potential route of SARS-CoV-2 transmission.^5^ However, studies related to the association between oral hygiene and COVID-19 infection are scarce. A very small-sample study (8 participants) indicated that viral shedding of SARS-CoV-2 was prolonged by around 15 days in patients with mental heath disorders who did not brush their teeth.^6^ In addition, the previous research examining the association between toothbrushing with similar respiratory diseases, such as pneumonia, showed that toothbrushing was associated with lower incidence, duration, and pneumonia mortality in community-dwelling individuals and hospitalised patients.^7^^,^^8^ A systematic review with meta-analysis concluded that the risk of ventilator-associated pneumonia was 24% lower in patients receiving chlorhexidine mouthwash combined with toothbrushing than in those receiving chlorhexidine mouthwash only.^9^ Also, some growing evidence from the industry of oral care products suggested that certain toothpastes containing zinc, stannous fluoride, or amine fluoride may play a role in temporarily reducing the viral load of SARS-CoV-2 intraorally.^10^ These laboratory studies suggested that these chemicals can render the SARS-CoV-2 virus non-infectious and prevent its multiplication in the host.^10^

This hypothesised microbial pathway for the association between toothbrushing habits and COVID-19 infection is not yet supported by enough evidence. Also, no epidemiologic study investigated this hypothesis. Hence, the aim of this epidemiologic study is to investigate the important public health question of whether the change in the time and the frequency of toothbrushing is associated with having the COVID-19 symptoms in a randomly sampled Japanese population.

## Methods

### Study design, setting, and participants

**Fig. 1:**
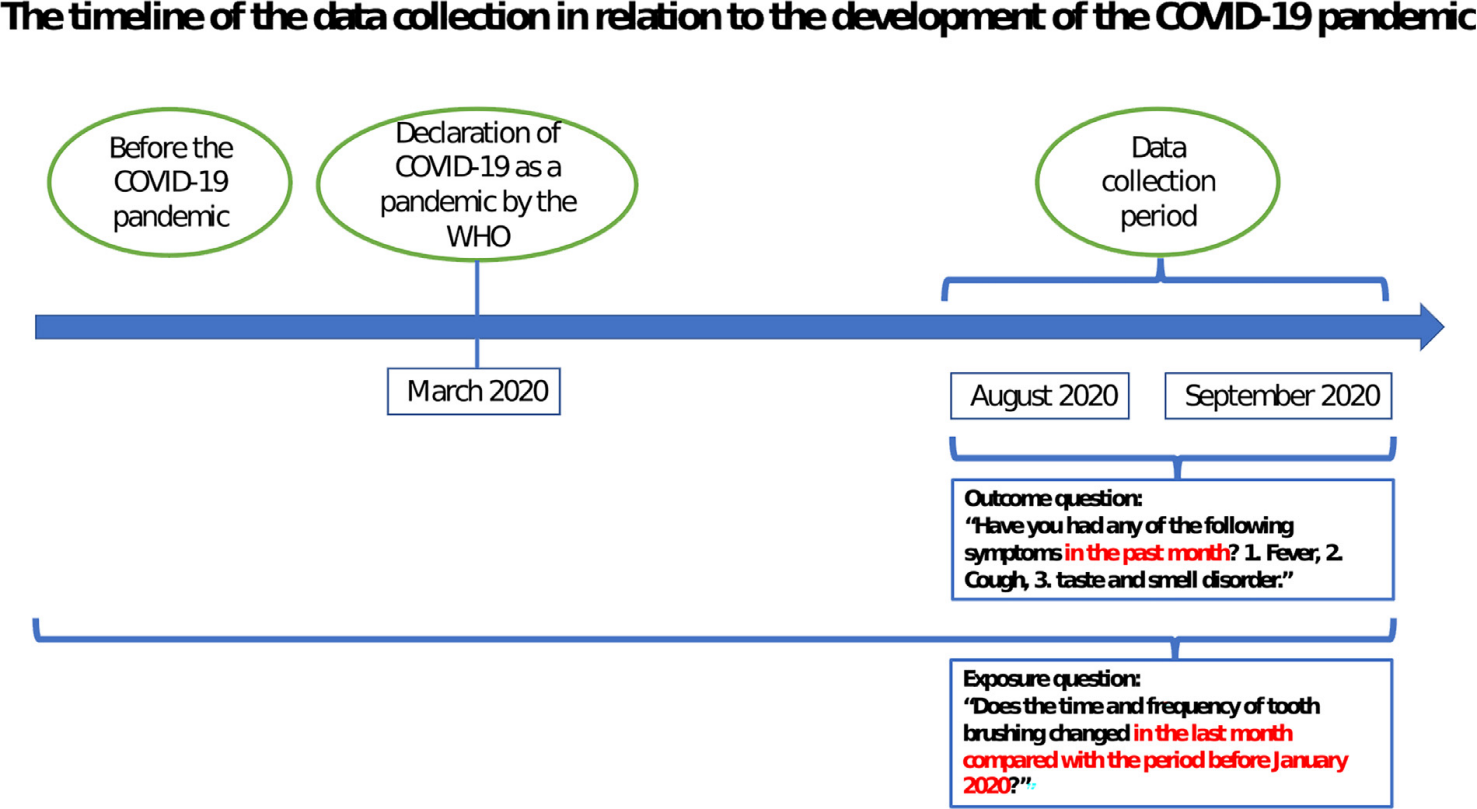
The timeline of the data collection showing the 8-month retrospective design of this study.

In this 8-month retrospective cohort study, we used data gathered between August 25, 2020, and September 30, 2020, from the Japan COVID-19 and Society Internet Survey (JACSIS), which targeted participants with a wide age range (from 15 to 79 years). Figure 1 illustrates the timeline of data gathering in relation to the development of the COVID-19 pandemic. The JACSIS is a self-reported internet survey conducted to assess the effects and the changes in the socioeconomic status, lifestyle, and health behaviours of the participants due to the COVID-19 pandemic.^11^ This internet survey was administered by a large internet research agency called Rakuten Insight, Inc., a Japanese online market research firm with approximately 2.3 million qualified Japanese respondents registered in their database as of March 2016.^12^ These individuals are users of the Rakuten Group services such as telecommunication services and online shopping.^13^ To compensate for the shortcomings of the online surveys, simple random sampling using a computer algorithm developed to select the target participants from the Rakuten Insight databases was conducted.^14^ This sampling was adjusted to match the population distribution in Japan by age, sex, and living area and covered all 47 prefectures of Japan using the respondents’ postal codes originally collected by Rakuten, Inc.^15^ The data from the National Survey on Living Standards, which is nationally representative of Japan, were used as a reference for the adjustment, weighting, and other processing schemes during the sampling.^11^^,^^15^ The survey, with informed consent at its beginning, was distributed to 224,389 individuals. When the predetermined response rate of 12.5% (28,000/224,389) was reached, the online survey was stopped.^15^ The survey design ensured that all survey questions must be answered. However, the participants could choose not to respond or to discontinue at any point during the survey. Those who stopped answering the survey were counted as nonrespondents. There were no missing values in the raw data set due to the described survey design. However, there was a possibility of inconsistent responses. Thus, we excluded 2386 participants because they showed inconsistent responses and were assumed to have answered the questionnaire haphazardly without reading it thoroughly (1955 participants failed to correctly answer a dummy question asking them to choose the second response from the bottom of a list of 5 available answers; 331 participants selected all items in a list of 7 substances in a question about drug abuse; and 100 participants selected all 16 diseases available in the list about existing comorbidities). The raw data of 25,614 respondents (91.4%) were used in this study. Of the 25,614 eligible participants, 3248 were excluded because they had only 1 or 2 of the 3 main symptoms of COVID-19 described below. The final sample size in this study was 22,366 respondents, 79.8% of the total survey respondents. Due to the JACSIS data usage and sharing policies, the data that support the findings of this study are available from the corresponding author upon reasonable request.

### Outcomes

According to the publicly available information from the National Health Services (NHS) in the UK, the 3 main symptoms of COVID-19 are high fever, cough, and taste and smell disorder.^16^ In the questionnaire, the participants were asked “Have you had any of the following symptoms in the past month? (1) Fever, (2) Cough, (3) Taste and Smell Disorder. The answers were binary “yes/no.” Having the 3 main COVID-19 symptoms altogether was used as a proxy for being infected with COVID-19.

### Exposures

The change in the time and the frequency of toothbrushing before and after the COVID-19 pandemic was used as an independent variable. This was a self-reported retrospective question as follows: “Has the time and frequency of toothbrushing changed in the last month compared with the period before January 2020?” There were 3 available choices: increased, same as before, and decreased. At first, the trichotomised variable was used to investigate the increase and the decrease in the time and the frequency of toothbrushing. Then, we dichotomised the responses into a binary variable (changed and unchanged) to examine the total effect of the change in the time and frequency of toothbrushing.

### Confounders

In this study, we adjusted for demographics, socioeconomic status (SES), general health status, health literacy, and living area. The age and sex of the participants were used as demographic confounders. The education attainment and the equivalised income level were used as socioeconomic determinants of health. The educational attainment was combined into 4 categories (high school or less, vocational school or college, university degree, and graduate school and others). The equivalised income level was converted from Japanese Yen to the United States Dollar (USD) and was calculated as the annual pretax household income divided by the square root of the number of people in the household and was categorised into (<25,000 USD/year, 25,000–45,000 USD/year, >45,000 USD/year, and do not want to answer or do not know). Self-rated health was used as an indicator of the general health status. It was categorised into good, fairly good, normal, not very good, and not good. For health literacy, we used the question “In the past month, did you refrain from going out unnecessarily or on a business trip?” The available answers were always, sometimes, almost never, and not at all. To account for the effects of regional variations in the rate of COVID-19 infections, the living area was adjusted in the fully adjusted model after being categorised into the following 7 living areas of Japan arranged from north to south (Hokkaido-Tohoku, Kanto, Hokuriku-Ko-shin-etsu, Tokai, Kansai, Chugoku-Shikoku, and Kyushu-Okinawa).

### Statistical analysis

**Fig. 2:**
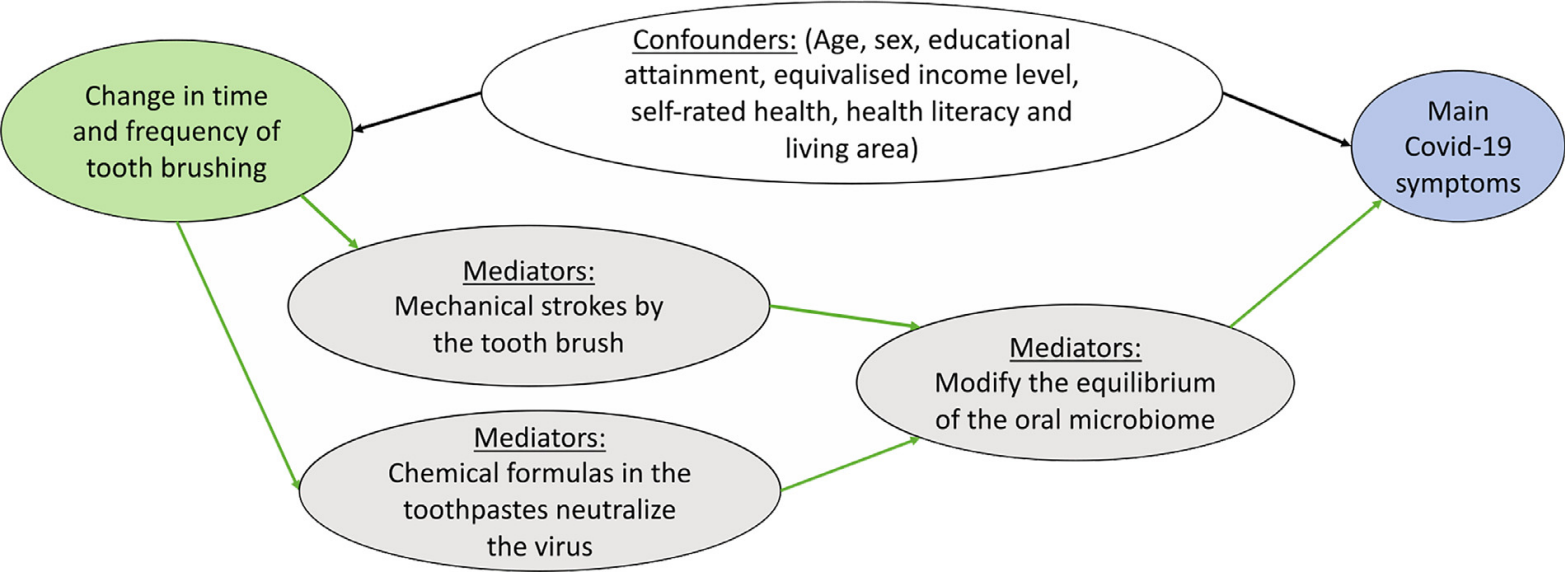
The hypothesised framework using the directed acyclic graph (DAG) for the association between the change in the time and the frequency of toothbrushing with the 3 main COVID-19 symptoms.

A descriptive analysis was performed to examine the characteristics of participants. Then, we used the logistic regression analyses to calculate the odds ratios (ORs) for having the 3 main COVID-19 symptoms altogether. For sensitivity analyses, we examined the association between the change in the time and frequency of toothbrushing with each of the 3 main COVID-19 symptoms as a separate outcome. As supplementary analyses, we examined the reverse association based on the hypothesis that COVID-19 infection led to changes in the time and frequency of toothbrushing after adjusting for the relevant confounders (the outcome, the change in the time and the frequency of toothbrushing, was trichotomised: 0 = decreased, 1 = unchanged, and 2 = increased). We used the directed acyclic graphs (DAGitty) version 3.0 to structure the hypothesised framework of this study (Figure 2).^17^ Stata 14 software from StataCorp LP was used for the analyses, and the STROBE guidelines for cohort studies were followed.

### Ethical approval

This study protocol was approved by the ethics committee at the Osaka International Cancer Institute, Japan (approval number: 20084).

## Results

Table 1 shows the characteristics of participants. From the 22,366 participants included in the analyses, the age range of the participants was 15 to 79 years (mean = 49.0 years, SD = ±17.3 years). In total, 11,014 participants were male (49.2%) and 11,352 were female (50.7%). Also, 2076 participants (9.3%) had increased time and frequency of toothbrushing, whilst 628 participants (2.8%) had decreased time and frequency of toothbrushing and 19,662 participants (87.9%) had unchanged time and frequency of toothbrushing. Only 60 participants (0.3%) had the 3 main COVID-19 symptoms. The proportions of those who had the 3 main COVID-19 symptoms was the highest amongst those with decreased time and frequency of toothbrushing, the youngest age group (15–29 years old), males, the group with the highest educational attainment (graduate school and other), the lowest income group (<25,000 USD/year), the group with the lowest self-rated health, those who almost never refrained from going outside unnecessarily in the past month, and those living in the Kansai area.

**Table 1:** The descriptive statistics of the participants and their stratification by having the 3 main COVID-19 symptoms (N = 22,366).

			Having the 3 main COVID-19 symptoms			
	Total		No		Yes	
	No.	%	No.	%	No.	%
**The change in time and frequency of toothbrushing**						
Increased	2076	9.3	2058	99.1	18	0.9
Unchanged	19,662	87.9	19,629	99.8	33	0.2
Decreased	628	2.8	619	98.6	9	1.4
**Age, y**						
15–29	3817	17.1	3789	99.3	28	0.7
30–49	7499	33.5	7477	99.7	22	0.3
50–59	3758	16.8	3755	99.9	3	0.1
60–79	7292	32.6	7285	99.9	7	0.1
**Sex**						
Male	11,014	49.2	10,974	99.6	40	0.4
Female	11,352	50.8	11,332	99.8	20	0.2
**Education attainment**						
High school or less	6656	29.8	6642	99.8	14	0.2
Vocational school or college	4957	22.2	4947	99.8	10	0.2
University degree	9614	43	9584	99.7	30	0.3
Graduate school and others	1139	5.1	1133	99.5	6	0.5
**Equivalised income level, USD/y**						
<25,000	4628	20.7	4606	99.5	22	0.5
25,000–45,000	7301	32.6	7284	99.8	17	0.2
>45,000	5738	25.7	5722	99.7	16	0.3
Do not want to answer or do not know	4699	21	4694	99.9	5	0.1
**Self-rated health**						
Good	4618	20.6	4604	99.7	14	0.3
Fairly good	7188	32.1	7180	99.9	8	0.1
Normal	7858	35.1	7846	99.8	12	0.2
Not very good	2209	9.9	2191	99.2	18	0.8
Not good	493	2.2	485	98.4	8	1.6
**Health literacy (refrain from going out unnecessarily)**						
Always	13,612	60.9	13,585	99.8	27	0.2
Sometimes	6453	28.9	6429	99.6	24	0.4
Almost never	1312	5.9	1306	99.5	6	0.5
Not at all	989	4.4	986	99.7	3	0.3
**Living areas**						
Hokkaido and Tohoku	2424	10.8	2421	99.9	3	0.1
Kanto	7930	35.5	7912	99.8	18	0.2
Hokuriku Ko-shin-etsu	2017	9.0	2012	99.8	5	0.2
Tokai	2025	9.1	2018	99.7	7	0.3
Kansai	3702	16.6	3682	99.5	20	0.5
Chugoku and Shikoku	1866	8.3	1865	99.9	1	0.1
Kyushu and Okinawa	2402	10.7	2396	99.8	6	0.2
**Total**	**22,366**	**100**	**22,306**	**99.7**	**60**	**0.3**

Table 2 shows the findings of the logistic regression analyses for the trichotomised outcome. All models showed that those who had increased or decreased time and frequency of toothbrushing had high odds of having the 3 main COVID-19 symptoms compared to those who had unchanged time and frequency of toothbrushing. However, the values of the ORs for those who had decreased time and frequency of toothbrushing were consistently higher than those who had increased time and frequency of toothbrushing in all models except the fully adjusted model.

**Table 2:** The findings of the logistic regression analyses for the association between the trichotomised change in the time and the frequency of toothbrushing with the 3 main COVID-19 symptoms (N = 22,366).

	Crude model (1)			Model (2) adjusted for age and sex			Model (3) adjusted for age, sex, and SES			Fully adjusted model (4)		
	Odds ratio	95% CI		Odds ratio	95% CI		Odds ratio	95% CI		Odds ratio	95% CI	
**Time and frequency of toothbrushing**												
Unchanged	**Reference**			**Reference**			**Reference**			**Reference**		
Increased	5.20	2.92	9.26	4.56	2.54	8.21	4.52	2.51	8.15	4.19	2.31	7.61
Decreased	8.65	4.12	18.15	5.48	2.57	11.73	5.13	2.39	11.04	3.83	1.71	8.59
**Age, y**												
15–29				**Reference**			**Reference**			**Reference**		
30–49				0.50	0.28	0.89	0.50	0.28	0.90	0.48	0.27	0.88
50–59				0.14	0.04	0.48	0.15	0.05	0.52	0.14	0.04	0.49
60–79				0.18	0.08	0.42	0.18	0.08	0.42	0.20	0.09	0.48
**Sex**												
Male				**Reference**			**Reference**			**Reference**		
Female				0.44	0.26	0.76	0.47	0.27	0.82	0.49	0.28	0.87
**Education attainment**												
High school or less							**Reference**			**Reference**		
Vocational school or college							1.08*	0.47	2.47	1.12*	0.49	2.58
University degree							1.13*	0.59	2.16	1.23*	0.64	2.38
Graduate school and others							1.88*	0.70	5.05	2.09*	0.77	5.66
**Equivalised income level, USD/y**												
<25,000							**Reference**			**Reference**		
25,000–45,000							0.53*	0.28	1.02	0.59*	0.31	1.14
>45,000							0.54*	0.28	1.06	0.61*	0.31	1.20
Do not want to answer or do not know							0.25	0.09	0.65	0.26	0.10	0.70
**Self-rated health**												
Good										**Reference**		
Fairly good										0.39	0.16	0.93
Normal										0.64*	0.29	1.40
Not very good										3.22	1.56	6.64
Not good										3.87	1.51	9.93
**Health literacy (refrain from going out unnecessarily)**												
Always										**Reference**		
Sometimes										1.59*	0.90	2.80
Almost never										1.40*	0.56	3.50
Not at all										0.69*	0.20	2.46
**Living area**												
Hokkaido and Tohoku										**Reference**		
Kanto										1.60*	0.46	5.49
Hokuriku Ko-shin-etsu										1.87*	0.44	7.95
Tokai										2.67*	0.68	10.45
Kansai										4.22*	1.24	14.40
Chugoku and Shikoku										0.41*	0.04	3.96
Kyushu and Okinawa										1.81*	0.45	7.30

SES, socioeconomic status.

Model 4 is adjusted for all confounders: age, sex, educational attainment, equivalised income level, self-rated health, health literacy, and living area.

All *P* values were <.05, except those with the asterisks, which were >.05.

Table 3 shows the findings of the logistic regression analyses for the dichotomised outcome (unchanged vs changed time and frequency of toothbrushing). All models showed that those who had a change in time and frequency of toothbrushing had higher ORs of having the 3 main COVID-19 symptoms compared to those who had unchanged toothbrushing habits. The ORs ranged from 6.00 (95% confidence interval [CI], 3.60–9.99; *P* < .001) in the crude model to 4.08 (95% CI, 2.38–6.98; *P* < .001) in the fully adjusted model.

**Table 3:** The findings of the logistic regression analyses for the association between the dichotomised change in the time and the frequency of toothbrushing with the three main COVID-19 symptoms (n=22,366)

	Crude model (1)			Model (2) adjusted for age and sex			Model (3) adjusted for age, sex, and SES			Fully adjusted model (4)		
	Odds ratio	95% CI		Odds ratio	95% CI		Odds ratio	95% CI		Odds ratio	95% CI	
**Time and frequency of toothbrushing**												
Unchanged	**Reference**			**Reference**			**Reference**			**Reference**		
Changed (increased and decreased)	6.00	3.60	9.99	4.83	2.86	8.16	4.70	2.78	7.96	4.08	2.38	6.98
**Age, y**												
15–29				**Reference**			**Reference**			**Reference**		
30–49				0.50	0.28	0.88	0.50	0.28	0.89	0.49	0.27	0.88
50–59				0.14	0.04	0.47	0.15	0.05	0.51	0.15	0.04	0.49
60–79				0.18	0.08	0.41	0.18	0.08	0.41	0.20	0.09	0.49
**Sex**												
Male				**Reference**			**Reference**			**Reference**		
Female				0.44	0.26	0.75	0.47	0.27	0.82	0.49	0.28	0.87
**Education attainment**												
High school or less							**Reference**			**Reference**		
Vocational school or college							1.08*	0.47	2.47	1.12*	0.49	2.58
University degree							1.13*	0.59	2.16	1.23*	0.64	2.38
Graduate school and others							1.87*	0.70	5.02	2.10*	0.77	5.68
**Equivalised income level, USD/y**												
<25,000							**Reference**			**Reference**		
25,000–45,000							0.53*	0.28	1.00	0.59*	0.31	1.15
>45,000							0.53*	0.27	1.05	0.61*	0.31	1.21
Do not want to answer or do not know							0.24	0.09	0.65	0.26	0.10	0.70
**Self-rated health**												
Good										**Reference**		
Fairly good										0.39	0.16	0.93
Normal										0.64*	0.29	1.39
Not very good										3.20	1.56	6.58
Not good										3.82	1.50	9.73
**Health literacy (refrain from going out unnecessarily)**												
Always										**Reference**		
Sometimes										1.58*	0.90	2.78
Almost never										1.38*	0.55	3.45
Not at all										0.68*	0.19	2.38
**Living area**												
Hokkaido and Tohoku										**Reference**		
Kanto										1.59*	0.46	5.48
Hokuriku Ko-shin-etsu										1.87*	0.44	7.95
Tokai										2.67*	0.68	10.47
Kansai										4.24	1.24	14.44
Chugoku and Shikoku										0.41*	0.04	3.94
Kyushu and Okinawa										1.81*	0.45	7.30

SES, socioeconomic status.

Model 4 is adjusted for all confounders: age, sex, educational attainment, equivalised income level, self-rated health, health literacy, and living area.

All *P* values were <.05, except those with asterisks, which were >.05.

The sensitivity analyses (Supplementary Tables 2–4) showed similar patterns compared to the main analyses for having high fever and taste and smell disorder as separate outcomes in all models. Having cough as an outcome was associated with the change in toothbrushing habits in all models except for those with decreased time and frequency of toothbrushing in the fully adjusted model.

Supplementary Table 1 shows the findings of the reverse association. Those who had the main COVID-19 symptoms had consistently higher odds of increasing the time and frequency of their toothbrushing in all 4 ordered logistic regression models when compared to those who did not have the main COVID-19 symptoms; the OR ranged from 2.25 (95% CI, 1.14–4.46) in the crude model to 2.52 (95% CI, 1.27–5.03) in the fully adjusted model.

## Discussion

To the best of our knowledge, this is the first epidemiologic study to examine the association between the change in the time and the frequency of toothbrushing and having the main COVID-19 symptoms. Compared to before the COVID-19 pandemic, both the increase and the decrease in the time and frequency of toothbrushing after the pandemic were associated with having the 3 main COVID-19 symptoms.

Our findings are consistent with the scarce evidence from previous studies which reported that decreased toothbrushing was associated with prolonged viral shedding of SARS-CoV-2.^6^ The disruption of the equilibrium of the oral microbiome could be a possible explanation of the association between the decrease in the time and the frequency of toothbrushing and having the 3 main COVID-19 symptoms. Reverse causation could explain the association between the increase in the time and the frequency of toothbrushing and having the 3 main COVID-19 symptoms (Supplementary Table 1). To explain these pathways in detail, it is possible that those who decreased the time and the frequency of toothbrushing could not have benefitted from the mechanical cleansing effect of toothbrushing and the protective antiviral effect of the chemicals in the toothpaste and had higher SARS-CoV-2 viral load intraorally and eventually had higher chances of having the 3 main COVID-19 symptoms.^6^, ^7^, ^8^, ^9^, ^10^ Figure 2 illustrates this hypothesised microbial pathway. For reverse causation, it is possible that those who had the 3 main COVID-19 symptoms became more health-cautious out of fear and panic after being infected with a new disease such as COVID-19 and started to brush their teeth more as a protective mechanism. Supplementary Figure 1 illustrates this pathway.

Our findings should be interpreted with caution. First, the inherent self-reported nature of our outcome as a proxy for COVID-19 infection was not supported by a clinical or a laboratory diagnosis. However as we followed a conservative approach of using the 3 main COVID-19 symptoms identified by the NHS it is likely that our cohort had contracted the illness. Second, we could not account for the asymptomatic COVID-19 cases, the cases with only 2 symptoms, or the cases with symptoms other than the 3 main symptoms used in this study. However, the sensitivity analyses using high fever, cough, and taste and smell disorder as separate outcomes showed similar patterns for the magnitude and the direction of the association with the change in time and frequency of toothbrushing. In addition, we used the self-reported measurements, which cause nondifferential misclassification. In this context, there is a possibility that the prevalence of the COVID-19 symptoms in this study did not reflect the actual situation. In fact, this study's prevalence for having the 3 main COVID-19 symptoms (0.3%) was much lower than the national average cumulative confirmed cases at the time of JACSIS data collection (1.6%), which was calculated from the national statistics.^18^^,^^19^ However, this misclassification was expected to occur equally in all toothbrushing categories which, in turn, led to the underestimation of the association between the exposure and the outcome.^20^ Third, although our study sample is a relatively large and the respondents were recruited to represent the Japanese population in terms of age, sex, and residential area, the generalisability of these findings is limited. This is because the respondents to a web-based survey might not fully represent the Japanese population. Fourth, an exact measurable change in the time and the frequency of toothbrushing could not be measured in this study due to data limitations. However, the proportion of those who brushed their teeth twice a day in a population-based study in Japan was 79.1% in 2017.^21^ This prevalence was proportional to those who reported unchanged time and frequency of toothbrushing in this study (87.9%). This implies that those who reported an increased or decreased time and frequency of toothbrushing either brush their teeth more than 2 times a day or fewer than 2 times a day, respectively.

On the other hand, and as strength points, laboratory studies and randomised controlled trials take time and might be infeasible to investigate the research question of this study. This is where the importance of this epidemiologic study is found: providing evidence regarding this association. Also, we used a relatively large data set, which improves the statistical power and the precision of the estimates. In addition, we adjusted for a wide range of possible confounders, which improves the robustness of the findings.

## Public health implications

This epidemiologic study raises the argument that there might be a microbial pathway explaining the association between toothbrushing and COVID-19 infection. Also, it suggests that maintaining toothbrushing habits at an appropriate time and frequency, for example, twice a day based on the consensus recommendation of the American Dental Association,^22^ might have a possible protective mechanism against COVID-19 infection. In relation to this, toothbrushing patterns are generally known to be a lifestyle habit that does not change much; however, 12.1% of the study participants changed their toothbrushing habits, and this is not considered to be a small percentage in the public health field. Besides, the findings of this study need to be confirmed by further research in the future including randomised controlled trials and cohort studies using clinical and/or laboratory data. In addition, similar cross-national comparative epidemiologic studies would provide better insights about the association between oral hygiene measures and COVID-19 infection in different countries.

## Conclusions

The change (decrease and increase) in time and frequency of toothbrushing from before to after the COVID-19 pandemic was associated with having the 3 main COVID-19 symptoms. It is possible that those who decreased their toothbrushing habits might not have benefitted from the cleansing and antiviral effects of regular toothbrushing and thus had higher SARS-CoV-2 viral load intraorally and eventually had higher chances of having the 3 main COVID-19 symptoms. Those who had the 3 main COVID-19 symptoms might have increased their toothbrushing habits out of fear and panic after being infected with COVID-19.

## Author contributions

Hazem Abbas contributed to the conception of the study, study design, data acquisition, data analyses, interpretation of the findings and drafted the manuscript; Kenji Takeuchi contributed to the conception, design, interpretation and editing the manuscript; Shihoko Koyama contributed to the study design, data aquisition and editing the manuscript;Ken Osaka contributed to the conception of the studyand editing the manuscript; Takahiro Tabuchi contributed to the data acquisition and editing the manuscript. All authors critically revised the manuscript, gave final approval and agree to be accountable for all aspects of the work ensuring integrity and accuracy.

## Conflict of interest

None disclosed.

## Appendix Supplementary materials

Image, application 1
