# Supplementary material for: Association Between Toothbrushing Habits and COVID-19 Symptoms
Source: Int Dent J. 2022 Aug 4;73(2):302–10. doi: 10.1016/j.identj.2022.07.011 (PMC9350673; doi:10.1016/j.identj.2022.07.011)
Supplement: Supplementary file 1 [file mmc1.docx]

**Supplementary table 1. The findings of the ordered logistic regression analyses for the reverse association between the three main covid-19 symptoms and the trichotomized change in the time and the frequency of tooth brushing with (n=22,366)**

|  | **Crude model (1)** | | | **Model (2) Adjusted for Age & Sex** | | | **Model (3) Adjusted for Age, Sex & SES** | | | **Fully adjusted model (4)** | | |
| --- | --- | --- | --- | --- | --- | --- | --- | --- | --- | --- | --- | --- |
|  | **Odds ratio** | 95%CI | | **Odds ratio** | 95%CI | | **Odds ratio** | 95%CI | | **Odds ratio** | 95%CI | |
| **The three main covid-19 symptoms** |  |  |  |  |  |  |  |  |  |  |  |  |
| Do not have | **Reference** |  |  | **Reference** |  |  | **Reference** |  |  | **Reference** |  |  |
| Have | 2.25 | 1.14 | 4.46 | 2.28 | 1.15 | 4.52 | 2.31 | 1.16 | 4.59 | 2.52 | 1.27 | 5.03 |
| **Age** |  |  |  |  |  |  |  |  |  |  |  |  |
| 15-29 years |  |  |  | **Reference** |  |  | **Reference** |  |  | **Reference** |  |  |
| 30-49 years |  |  |  | 0.72 | 0.64 | 0.81 | 0.70 | 0.62 | 0.79 | 0.69 | 0.61 | 0.78 |
| 50-59 years |  |  |  | 0.77 | 0.67 | 0.88 | 0.75 | 0.65 | 0.86 | 0.74 | 0.64 | 0.85 |
| 60-79 years |  |  |  | 0.85 | 0.75 | 0.95 | 0.86 | 0.77 | 0.97 | 0.80 | 0.71 | 0.91 |
| **Sex** |  |  |  |  |  |  |  |  |  |  |  |  |
| Male |  |  |  | **Reference** |  |  | **Reference** |  |  | **Reference** |  |  |
| Female |  |  |  | 1.46 | 1.34 | 1.58 | 1.54 | 1.41 | 1.67 | 1.40 | 1.29 | 1.53 |
| **Education attainment** |  |  |  |  |  |  |  |  |  |  |  |  |
| High school or lower |  |  |  |  |  |  | **Reference** |  |  | **Reference** |  |  |
| vocational school or College |  |  |  |  |  |  | 1.00^†^ | 0.89 | 1.12 | 0.96^†^ | 0.86 | 1.08 |
| University degree |  |  |  |  |  |  | 1.11^†^ | 1.00 | 1.22 | 1.06^†^ | 0.96 | 1.17 |
| Graduate school and others |  |  |  |  |  |  | 1.12^†^ | 0.92 | 1.36 | 1.07^†^ | 0.88 | 1.31 |
| **Eqivalised income level** |  |  |  |  |  |  |  |  |  |  |  |  |
| <25000 $/year |  |  |  |  |  |  | **Reference** |  |  | **Reference** |  |  |
| 25000 - 45000 $/year |  |  |  |  |  |  | 1.29 | 1.14 | 1.44 | 1.22 | 1.09 | 1.37 |
| >45000 $/year |  |  |  |  |  |  | 1.42 | 1.26 | 1.61 | 1.34 | 1.18 | 1.51 |
| Do not want to answer or Do not know |  |  |  |  |  |  | 1.11^†^ | 0.98 | 1.26 | 1.09^†^ | 0.96 | 1.24 |
| **Self-rated health** |  |  |  |  |  |  |  |  |  |  |  |  |
| good |  |  |  |  |  |  |  |  |  | **Reference** |  |  |
| fairly good |  |  |  |  |  |  |  |  |  | 0.95^†^ | 0.85 | 1.06 |
| normal |  |  |  |  |  |  |  |  |  | 0.78 | 0.70 | 0.88 |
| not very good |  |  |  |  |  |  |  |  |  | 0.81 | 0.69 | 0.95 |
| not good |  |  |  |  |  |  |  |  |  | 0.37 | 0.27 | 0.50 |
| **Health literacy (refrain from going out unnecessarily)** |  |  |  |  |  |  |  |  |  |  |  |  |
| Always |  |  |  |  |  |  |  |  |  | **Reference** |  |  |
| Sometimes |  |  |  |  |  |  |  |  |  | 0.72 | 0.66 | 0.79 |
| Almost never |  |  |  |  |  |  |  |  |  | 0.49 | 0.41 | 0.60 |
| Not at all |  |  |  |  |  |  |  |  |  | 0.36 | 0.29 | 0.45 |
| **Living area** |  |  |  |  |  |  |  |  |  |  |  |  |
| Hokkaido and Tohoku |  |  |  |  |  |  |  |  |  | **Reference** |  |  |
| Kanto |  |  |  |  |  |  |  |  |  | 1.11^†^ | 0.96 | 1.28 |
| Hokuriku Ko-shin-etsu |  |  |  |  |  |  |  |  |  | 1.01^†^ | 0.84 | 1.21 |
| Tokai |  |  |  |  |  |  |  |  |  | 1.05^†^ | 0.87 | 1.26 |
| Kansai |  |  |  |  |  |  |  |  |  | 1.18 | 1.01 | 1.39 |
| Chugoku and Shikoku |  |  |  |  |  |  |  |  |  | 0.86^†^ | 0.71 | 1.04 |
| Kyushu and Okinawa |  |  |  |  |  |  |  |  |  | 1.19^†^ | 1.00 | 1.41 |

**Abbreviations; SES=socio-economic status.**

**Model 4 is adjusted for all confounders; age, sex, educational attainment, equivalized income level, self-rated health, health literacy and living area.**

**All p-values were <0.05, except those with the sign † p-values>0.05**

**Supplementary table 2. The findings of the logistic regression analyses for the association between the trichotomized change in the time and the frequency of tooth brushing with the having high fever (n=25,614)**

|  | **Crude model (1)** | | | **Model (2) adjusted for Age & Sex** | | | **Model (3) adjusted for Age, Sex & SES** | | | **Fully adjusted model (4)** | | |
| --- | --- | --- | --- | --- | --- | --- | --- | --- | --- | --- | --- | --- |
|  | **Odds ratio** | 95%CI | 95%CI | **Odds ratio** | 95%CI | 95%CI | **Odds ratio** | 95%CI | 95%CI | **Odds ratio** | 95%CI | 95%CI |
| **Time and Frequency of Tooth brushing** |  |  |  |  |  |  |  |  |  |  |  |  |
| Unchanged | **Reference** |  |  | **Reference** |  |  | **Reference** |  |  | **Reference** |  |  |
| Increased | 2.31 | 1.81 | 2.96 | 2.06 | 1.60 | 2.65 | 2.04 | 1.59 | 2.63 | 2.08 | 1.61 | 2.68 |
| Decreased | 4.04 | 2.90 | 5.62 | 2.76 | 1.96 | 3.86 | 2.68 | 1.91 | 3.76 | 2.22 | 1.57 | 3.15 |
| **Age** |  |  |  |  |  |  |  |  |  |  |  |  |
| 15-29 years |  |  |  | **Reference** |  |  | **Reference** |  |  | **Reference** |  |  |
| 30-49 years |  |  |  | 0.50 | 0.40 | 0.62 | 0.48 | 0.38 | 0.60 | 0.46 | 0.36 | 0.58 |
| 50-59 years |  |  |  | 0.32 | 0.23 | 0.44 | 0.31 | 0.22 | 0.43 | 0.29 | 0.21 | 0.40 |
| 60-79 years |  |  |  | 0.24 | 0.18 | 0.32 | 0.24 | 0.18 | 0.32 | 0.24 | 0.18 | 0.32 |
| **Sex** |  |  |  |  |  |  |  |  |  |  |  |  |
| Male |  |  |  | **Reference** |  |  | **Reference** |  |  | **Reference** |  |  |
| Female |  |  |  | 0.57 | 0.47 | 0.69 | 0.57 | 0.47 | 0.70 | 0.61 | 0.50 | 0.75 |
| **Education attainment** |  |  |  |  |  |  |  |  |  |  |  |  |
| High school or lower |  |  |  |  |  |  | **Reference** |  |  | **Reference** |  |  |
| Vocational school or College |  |  |  |  |  |  | 1.21^†^ | 0.91 | 1.59 | 1.23^†^ | 0.93 | 1.62 |
| University degree |  |  |  |  |  |  | 0.96^†^ | 0.76 | 1.21 | 0.99^†^ | 0.78 | 1.26 |
| Graduate school and others |  |  |  |  |  |  | 1.16^†^ | 0.77 | 1.75 | 1.17^†^ | 0.78 | 1.78 |
| **Equavilsed income level** |  |  |  |  |  |  |  |  |  |  |  |  |
| <25000 $/year |  |  |  |  |  |  | **Reference** |  |  | **Reference** |  |  |
| 25000 - 45000 $/year |  |  |  |  |  |  | 0.75 | 0.58 | 0.96 | 0.81^†^ | 0.63 | 1.04 |
| >45000 $/year |  |  |  |  |  |  | 0.86^†^ | 0.67 | 1.12 | 0.95^†^ | 0.73 | 1.23 |
| Do not want to answer or Do not know |  |  |  |  |  |  | 0.52 | 0.38 | 0.71 | 0.55 | 0.40 | 0.76 |
| **Self-rated health** |  |  |  |  |  |  |  |  |  |  |  |  |
| Good |  |  |  |  |  |  |  |  |  | **Reference** |  |  |
| Fairly good |  |  |  |  |  |  |  |  |  | 1.54 | 1.13 | 2.11 |
| Normal |  |  |  |  |  |  |  |  |  | 1.51 | 1.11 | 2.07 |
| Not very good |  |  |  |  |  |  |  |  |  | 2.91 | 2.07 | 4.10 |
| Not good |  |  |  |  |  |  |  |  |  | 2.98 | 1.84 | 4.84 |
| **Health literacy (refrain from going out unnecessarily)** |  |  |  |  |  |  |  |  |  |  |  |  |
| Always |  |  |  |  |  |  |  |  |  | **Reference** |  |  |
| Sometimes |  |  |  |  |  |  |  |  |  | 1.31 | 1.06 | 1.62 |
| Almost never |  |  |  |  |  |  |  |  |  | 1.85 | 1.35 | 2.53 |
| Not at all |  |  |  |  |  |  |  |  |  | 1.15^†^ | 0.74 | 1.78 |
| **Living area** |  |  |  |  |  |  |  |  |  |  |  |  |
| Hokkaido and Tohoku |  |  |  |  |  |  |  |  |  | **Reference** |  |  |
| Kanto |  |  |  |  |  |  |  |  |  | 1.01^†^ | 0.71 | 1.43 |
| Hokuriku Ko-shin-etsu |  |  |  |  |  |  |  |  |  | 1.17^†^ | 0.76 | 1.81 |
| Tokai |  |  |  |  |  |  |  |  |  | 1.23^†^ | 0.80 | 1.88 |
| Kansai |  |  |  |  |  |  |  |  |  | 1.16^†^ | 0.79 | 1.69 |
| Chugoku and Shikoku |  |  |  |  |  |  |  |  |  | 1.31^†^ | 0.85 | 2.01 |
| Kyushu and Okinawa |  |  |  |  |  |  |  |  |  | 1.00^†^ | 0.65 | 1.53 |

**Abbreviations; SES=socio-economic status.**

**Model 4 is adjusted for all confounders; age, sex, educational attainment, equivalized income level, self-rated health, health literacy and living area.**

**All p-values were <0.05, except those with the sign † p-values>0.05**

**Supplementary table 3. The findings of the logistic regression analyses for the association between the trichotomized change in the time and the frequency of tooth brushing with the having cough (n=25,614)**

|  | **Crude model (1)** | | | **Model (2) adjusted for Age & Sex** | | | **Model (3) adjusted for Age, Sex & SES** | | | **Fully adjusted model (4)** | | |
| --- | --- | --- | --- | --- | --- | --- | --- | --- | --- | --- | --- | --- |
|  | **Odds ratio** | 95%CI | 95%CI | **Odds ratio** | 95%CI | 95%CI | **Odds ratio** | 95%CI | 95%CI | **Odds ratio** | 95%CI | 95%CI |
| **Time and Frequency of Tooth brushing** |  |  |  |  |  |  |  |  |  |  |  |  |
| Unchanged | **Reference** |  |  | **Reference** |  |  | **Reference** |  |  | **Reference** |  |  |
| Increased | 1.42 | 1.27 | 1.60 | 1.45 | 1.29 | 1.63 | 1.46 | 1.30 | 1.65 | 1.46 | 1.29 | 1.64 |
| Decreased | 1.28 | 1.04 | 1.58 | 1.25 | 1.01 | 1.55 | 1.23^†^ | 0.99 | 1.52 | 1.03^†^ | 0.83 | 1.28 |
| **Age** |  |  |  |  |  |  |  |  |  |  |  |  |
| 15-29 years |  |  |  | **Reference** |  |  | **Reference** |  |  | **Reference** |  |  |
| 30-49 years |  |  |  | 1.19 | 1.06 | 1.33 | 1.20 | 1.07 | 1.34 | 1.10^†^ | 0.98 | 1.23 |
| 50-59 years |  |  |  | 1.02^†^ | 0.89 | 1.17 | 1.04^†^ | 0.91 | 1.19 | 0.91^†^ | 0.80 | 1.05 |
| 60-79 years |  |  |  | 0.93^†^ | 0.83 | 1.05 | 0.91^†^ | 0.81 | 1.03 | 0.86 | 0.76 | 0.98 |
| **Sex** |  |  |  |  |  |  |  |  |  |  |  |  |
| Male |  |  |  | **Reference** |  |  | **Reference** |  |  | **Reference** |  |  |
| Female |  |  |  | 0.89 | 0.82 | 0.96 | 0.87 | 0.80 | 0.94 | 0.90 | 0.83 | 0.98 |
| **Education attainment** |  |  |  |  |  |  |  |  |  |  |  |  |
| High school or lower |  |  |  |  |  |  | **Reference** |  |  | **Reference** |  |  |
| Vocational school or College |  |  |  |  |  |  | 0.99^†^ | 0.89 | 1.11 | 1.02^†^ | 0.92 | 1.14 |
| University degree |  |  |  |  |  |  | 0.94^†^ | 0.85 | 1.03 | 0.99^†^ | 0.90 | 1.09 |
| Graduate school and others |  |  |  |  |  |  | 0.94^†^ | 0.78 | 1.13 | 0.98^†^ | 0.81 | 1.18 |
| **Equavilsed income level** |  |  |  |  |  |  |  |  |  |  |  |  |
| <25000 $/year |  |  |  |  |  |  | **Reference** |  |  | **Reference** |  |  |
| 25000 - 45000 $/year |  |  |  |  |  |  | 0.82 | 0.74 | 0.91 | 0.90 | 0.81 | 1.00 |
| >45000 $/year |  |  |  |  |  |  | 0.73 | 0.65 | 0.82 | 0.81 | 0.72 | 0.91 |
| Do not want to answer or Do not know |  |  |  |  |  |  | 0.74 | 0.66 | 0.84 | 0.79 | 0.70 | 0.89 |
| **Self-rated health** |  |  |  |  |  |  |  |  |  |  |  |  |
| Good |  |  |  |  |  |  |  |  |  | **Reference** |  |  |
| Fairly good |  |  |  |  |  |  |  |  |  | 1.76 | 1.54 | 2.02 |
| Normal |  |  |  |  |  |  |  |  |  | 1.97 | 1.73 | 2.26 |
| Not very good |  |  |  |  |  |  |  |  |  | 4.58 | 3.96 | 5.30 |
| Not good |  |  |  |  |  |  |  |  |  | 3.97 | 3.16 | 4.97 |
| **Health literacy (refrain from going out unnecessarily)** |  |  |  |  |  |  |  |  |  |  |  |  |
| Always |  |  |  |  |  |  |  |  |  | **Reference** |  |  |
| Sometimes |  |  |  |  |  |  |  |  |  | 1.10 | 1.01 | 1.20 |
| Almost never |  |  |  |  |  |  |  |  |  | 1.21 | 1.03 | 1.41 |
| Not at all |  |  |  |  |  |  |  |  |  | 0.90^†^ | 0.74 | 1.10 |
| **Living area** |  |  |  |  |  |  |  |  |  |  |  |  |
| Hokkaido and Tohoku |  |  |  |  |  |  |  |  |  | **Reference** |  |  |
| Kanto |  |  |  |  |  |  |  |  |  | 0.94^†^ | 0.82 | 1.07 |
| Hokuriku Ko-shin-etsu |  |  |  |  |  |  |  |  |  | 0.98^†^ | 0.82 | 1.16 |
| Tokai |  |  |  |  |  |  |  |  |  | 0.91^†^ | 0.77 | 1.09 |
| Kansai |  |  |  |  |  |  |  |  |  | 0.88^†^ | 0.75 | 1.02 |
| Chugoku and Shikoku |  |  |  |  |  |  |  |  |  | 0.94^†^ | 0.79 | 1.13 |
| Kyushu and Okinawa |  |  |  |  |  |  |  |  |  | 1.08^†^ | 0.92 | 1.27 |

**Abbreviations; SES=socio-economic status.**

**Model 4 is adjusted for all confounders; age, sex, educational attainment, equivalized income level, self-rated health, health literacy and living area.**

**All p-values were <0.05, except those with the sign † p-values>0.05**

**Supplementary table 4. The findings of the logistic regression analyses for the association between the trichotomized change in the time and the frequency of tooth brushing with the having taste and smell disorder (n=25,614)**

|  | **Crude model (1)** | | | **Model (2) adjusted for Age & Sex** | | | **Model (3) adjusted for Age, Sex & SES** | | | **Fully adjusted model (4)** | | |
| --- | --- | --- | --- | --- | --- | --- | --- | --- | --- | --- | --- | --- |
|  | **Odds ratio** | 95%CI | 95%CI | **Odds ratio** | 95%CI | 95%CI | **Odds ratio** | 95%CI | 95%CI | **Odds ratio** | 95%CI | 95%CI |
| **Time and Frequency of Tooth brushing** |  |  |  |  |  |  |  |  |  |  |  |  |
| Unchanged | **Reference** |  |  | **Reference** |  |  | **Reference** |  |  | **Reference** |  |  |
| Increased | 2.96 | 2.17 | 4.03 | 2.84 | 2.07 | 3.88 | 2.85 | 2.08 | 3.91 | 2.72 | 1.98 | 3.75 |
| Decreased | 5.13 | 3.41 | 7.71 | 4.12 | 2.71 | 6.26 | 3.89 | 2.56 | 5.93 | 2.62 | 1.69 | 4.07 |
| **Age** |  |  |  |  |  |  |  |  |  |  |  |  |
| 15-29 years |  |  |  | **Reference** |  |  | **Reference** |  |  | **Reference** |  |  |
| 30-49 years |  |  |  | 0.62 | 0.45 | 0.85 | 0.65 | 0.47 | 0.90 | 0.60 | 0.43 | 0.84 |
| 50-59 years |  |  |  | 0.62 | 0.41 | 0.92 | 0.66 | 0.44 | 0.99 | 0.59 | 0.39 | 0.90 |
| 60-79 years |  |  |  | 0.45 | 0.31 | 0.65 | 0.44 | 0.31 | 0.64 | 0.46 | 0.32 | 0.67 |
| **Sex** |  |  |  |  |  |  |  |  |  |  |  |  |
| Male |  |  |  | **Reference** |  |  | **Reference** |  |  | **Reference** |  |  |
| Female |  |  |  | 0.62 | 0.48 | 0.80 | 0.63 | 0.48 | 0.82 | 0.69 | 0.52 | 0.90 |
| **Education attainment** |  |  |  |  |  |  |  |  |  |  |  |  |
| High school or lower |  |  |  |  |  |  | **Reference** |  |  | **Reference** |  |  |
| Vocational school or College |  |  |  |  |  |  | 0.83^†^ | 0.57 | 1.22 | 0.87^†^ | 0.59 | 1.28 |
| University degree |  |  |  |  |  |  | 0.98^†^ | 0.73 | 1.32 | 1.07^†^ | 0.79 | 1.45 |
| Graduate school and others |  |  |  |  |  |  | 0.83^†^ | 0.44 | 1.54 | 0.84^†^ | 0.45 | 1.57 |
| **Equavilsed income level** |  |  |  |  |  |  |  |  |  |  |  |  |
| <25000 $/year |  |  |  |  |  |  | **Reference** |  |  | **Reference** |  |  |
| 25000 - 45000 $/year |  |  |  |  |  |  | 0.61 | 0.44 | 0.84 | 0.74^†^ | 0.53 | 1.03 |
| >45000 $/year |  |  |  |  |  |  | 0.58 | 0.41 | 0.83 | 0.73^†^ | 0.51 | 1.05 |
| Do not want to answer or Do not know |  |  |  |  |  |  | 0.49 | 0.33 | 0.73 | 0.54 | 0.36 | 0.81 |
| **Self-rated health** |  |  |  |  |  |  |  |  |  |  |  |  |
| Good |  |  |  |  |  |  |  |  |  | **Reference** |  |  |
| Fairly good |  |  |  |  |  |  |  |  |  | 0.81^†^ | 0.52 | 1.26 |
| Normal |  |  |  |  |  |  |  |  |  | 1.10^†^ | 0.73 | 1.67 |
| Not very good |  |  |  |  |  |  |  |  |  | 3.45 | 2.27 | 5.23 |
| Not good |  |  |  |  |  |  |  |  |  | 6.38 | 3.89 | 10.46 |
| **Health literacy (refrain from going out unnecessarily)** |  |  |  |  |  |  |  |  |  |  |  |  |
| Always |  |  |  |  |  |  |  |  |  | **Reference** |  |  |
| Sometimes |  |  |  |  |  |  |  |  |  | 0.95^†^ | 0.70 | 1.28 |
| Almost never |  |  |  |  |  |  |  |  |  | 1.64 | 1.07 | 2.49 |
| Not at all |  |  |  |  |  |  |  |  |  | 1.32^†^ | 0.80 | 2.16 |
| **Living area** |  |  |  |  |  |  |  |  |  |  |  |  |
| Hokkaido and Tohoku |  |  |  |  |  |  |  |  |  | **Reference** |  |  |
| Kanto |  |  |  |  |  |  |  |  |  | 0.94^†^ | 0.60 | 1.48 |
| Hokuriku Ko-shin-etsu |  |  |  |  |  |  |  |  |  | 1.17^†^ | 0.66 | 2.05 |
| Tokai |  |  |  |  |  |  |  |  |  | 0.98^†^ | 0.55 | 1.76 |
| Kansai |  |  |  |  |  |  |  |  |  | 1.50^†^ | 0.94 | 2.40 |
| Chugoku and Shikoku |  |  |  |  |  |  |  |  |  | 0.74^†^ | 0.39 | 1.41 |
| Kyushu and Okinawa |  |  |  |  |  |  |  |  |  | 0.94^†^ | 0.54 | 1.64 |

**Abbreviations; SES=socio-economic status.**

**Model 4 is adjusted for all confounders; age, sex, educational attainment, equivalized income level, self-rated health, health literacy and living area.**

**All p-values were <0.05, except those with the sign † p-values>0.05**

**Supplementary figure 1. The hypothesized framework using the directed acyclic graph (DAG) for the reverse association between having the three main covid-19 symptoms (exposure) and the change in the time and the frequency of tooth brushing (outcome).**
